# Analytical and clinical evaluation of a duplex RT-qPCR assay for the detection and identification of o’nyong-nyong and chikungunya virus

**DOI:** 10.1080/22221751.2024.2429650

**Published:** 2024-11-12

**Authors:** Konrad M. Wesselmann, Lea Luciani, Laurence Thirion, Xavier de Lamballerie, Remi Charrel, Laura Pezzi

**Affiliations:** a Unité des Virus Émergents (UVE: Aix-Marseille Univ, Università di Corsica, IRD 190, Inserm 1207, IRBA), Marseille, France; b Assistance publique-hôpitaux de Marseille (AP-HM), Service de virologie aiguë et tropicale, Marseille, France; c Centre National de Référence des Arbovirus, Inserm-IRBA, Marseille, France

**Keywords:** Alphavirus, togaviridae, arbovirus, Chikungunya virus, o’nyong-nyong virus, diagnostics, RT-qPCR

## Abstract

The mosquito-borne alphavirus o’nyong-nyong virus (ONNV) has proven its potential to cause major human outbreaks. On the African continent, ONNV causes unspecific febrile illness and co-circulates with the close relative chikungunya virus (CHIKV). The true scale of ONNV burden is poorly understood in Africa, because of the scarce availability of molecular in-house and commercial assays, strong cross-reactivity between ONNV and CHIKV in serological assays and a lack of surveillance. We designed a new RT-qPCR assay targeting the E1 gene for the detection of ONNV that can be used in monoplex or in duplex format, combined with a previously published CHIKV monoplex assay targeting the nsp1. The lower limit of detection with 95% positivity rate was determined to be <10 RNA copies/µL in monoplex and duplex format for both ONNV and CHIKV. Both monoplex assays and the duplex assay proved to be linear within the tested range of approximately 10^8^ to 10^2^ RNA copies/µl, and showed 100% specificity against a wide panel of arbovirus supernatants as well as several other pathogens in clinical samples. Testing of CHIKV positive serum and ONNV-spiked plasma samples confirmed the suitability of the assays in a clinical setting. The new assays provide a robust tool for molecular ONNV as well as ONNV/CHIKV simultaneous detection and may contribute to clarify the true burden of the two viruses, to improve arbovirus surveillance and to strengthen epidemics preparedness in Africa.

## Introduction

O’nyong-nyong virus (ONNV) (genus *Alphavirus*, family *Togaviridae)* is primarily transmitted by *Anopheles (An.) gambiae* and *An. funestus* mosquitoes in Africa [1]. First isolated in Uganda in 1959, ONNV has caused two major outbreaks in sub-Saharan countries [2,3], interspersed with sporadic detection in humans and vectors [4]. The true scale of ONNV circulation in Africa remains difficult to understand and is most probably underestimated, due to limited surveillance [5]. Although seroprevalence studies suggest continued transmission and human exposure to ONNV [6–9], scarce available data have often limited reliability due to strong cross-reactivity with antibodies raised against closely related alphaviruses, especially chikungunya virus (CHIKV) [10,11]. In addition to the close phylogenetic relationship, ONNV and CHIKV co-circulate in Africa and share an apparent similar clinical picture causing febrile disease and an arthritogenic syndrome [4,5]. The diagnosis in the acute phase must involve specific molecular biology tools to differentiate an infection by ONNV from an infection by CHIKV. The robustness of new molecular assays depends on the quality and availability of genomic data, which is currently limited for ONNV. A recent external quality assessment (EQA) of alphaviruses molecular detection carried out by EVD-Labnet involving European laboratories has shown that several PCR assays (in both species-specific and pan-alphavirus formats) may lack sensitivity in ONNV detection, or specificity (detecting ONNV on CHIKV-positive samples, or CHIKV on ONNV-positive samples) (in press). Since very few in-house PCR assays have been developed for ONNV [10] and because of the co-circulation of CHIKV and ONNV, a molecular biology tool capable of identifying the two viruses is therefore greatly needed by the medical and scientific communities. In this study, we describe a new real-time PCR (RT-qPCR) assay for the detection of ONNV that can be used as either a monoplex test or in a multiplex format with a CHIKV RT-qPCR assay.

## Materials and methods

### Design of ONNV monoplex RT-qPCR assay and development of ONNV-CHIKV duplex RT-qPCR assay

The NCBI GenBank (https://www.ncbi.nlm.nih.gov/genbank/) was searched for ONNV sequences using the search term “Onyong-nyong virus” (search performed on 08/02/2024). Of the 29 available sequences 14 were excluded (Table S1) for either not referring to genomic RNA (*n* = 12) or because they were identical to included sequences (*n* = 2). To design the ONNV monoplex RT-qPCR assay, all remaining partial (*n* = 7) and complete sequences (*n* = 8) (Table S2) were aligned using MAFFT (https://mafft.cbrc.jp/; algorithm: auto; scoring matrix BLOSUM62). Seventeen CHIKV complete genome sequences covering the five CHIKV lineages (Eastern/Central/Southern Africa-ECSA 1-3, West African and Asian) were added to the alignment (Table S2), in order to identify a target region that (i) was highly conserved among ONNV sequences and (ii) contained a sufficient number of mutations between ONNV and CHIKV. Seven strains of different alphaviruses, namely Una virus (UNAV), Sindbis virus (SINV), Semliki Forest virus (SFV), Ross River virus (RRV), Mayaro virus (MAYV), Bebaru virus (BEBV) and Getah virus (GETV), were also included in the alignment (Table S2) to verify the analytical specificity of the ONNV assay.

To develop the ONNV-CHIKV duplex RT-qPCR assay, a CHIKV monoplex assay was selected to be used in combination with the ONNV monoplex assay [12], here called “Panning CHIKV monoplex assay”. The Panning CHIKV monoplex assay consists of one hydrolysis probe (FAM-labelled) and two sets of forward and reverse primers targeting the non-structural protein-1 (nsp1) gene: one set of primers is adapted to all CHIKV lineages, whereas the second one is best suited for strains belonging to ECSA3 lineage [12]. When included in the ONNV-CHIKV duplex assay, the ONNV monoplex assay’s probe was VIC-labelled. Details of the ONNV RT-qPCR monoplex assay and ONNV-CHIKV duplex RT-qPCR assay are presented in Table 1.

**Table 1.** Primers and probes included in the ONNV-CHIKV duplex RT-qPCR assay.

Reference	Primer/probe	5’–> 3’ Sequence	Target gene	Position*	Amplicon size (nts)	Final concentration [nM]
In-house ONNV monoplex [developed in this study]	ONNV-F	GCTAAACTACGTGTCTTTTACCAAG	E1	10,484	21	333
	ONNV-R	GCAGGGAGGCCAGGACAGTTCGG		10,673		333
	ONNV-*P*	VIC-CCACTATCGTCCGCCTGGTCACCA-QSY		10,586		133
Panning et al. [12]	CHIKV-F1	TGATCCCGACTCAACCATCCT	nsp1	241	82	600
	CHIKV-R1	GGCAAACGCAGTGGTACTTCCT		302		600
	CHIKV-F2	CCGACTCAACCATCCTGGAT		246	77	600
	CHIKV-R2	GGCAGACGCAGTGGTACTTCCT		302		600
	CHIKV-*P*^$^	FAM-TCCGACATCATCCTCCTTGCTGGC-TAMRA		277		200

*Position refers to CHIKV strain 06113879 (GenBank accession number: MH229986.1) and ONNV (GenBank Accession number: M20303). ^$^TAMRA Quencher replaces BHQ1 from the original study. Nsp1: non-structural protein; nts: nucleotides.

### RT-qPCR

Supernatant of viral cell cultures and clinical samples used in this study were extracted using the EZ1 virus mini kit v2.0 on the BioRobot EZ1 Advanced XL (both from QIAGEN, Hilden, Germany). All RT-qPCR reactions described in this study were performed using SuperScript® III Platinum® One-Step RT-qPCR Kit with ROX (#11732-088, Invitrogen-Thermo Fisher Scientific, Waltham, MA, USA) on a BioRad CFX96TM thermal cycler, software version 3.1 (Bio-Rad Laboratories, Hercules, CA, USA). The final reaction mix had a volume of 25 µL consisting of 5 µL sample RNA, 12.5 µL 2x Reaction Mix, 0.5 µL of Superscript III RT/Platinum Taq Mix and primers and probes at concentrations described in Table 1. Cycling conditions were: 50°C for 15 min; 95°C for 2 min; 45 cycles of 95°C for 15 s and 60°C for 45 s. Positive and negative controls were included in each PCR run.

### Limit of detection

The limits of detection of the ONNV monoplex assay and the ONNV-CHIKV duplex assay were evaluated using one ONNV strain and five CHIKV strains representing the five CHIKV lineages (listed in Table 2). Genomic RNA was obtained from the freeze-dried cell culture supernatant and serially diluted. Eight serially decreasing dilutions of each strain with concentrations ranging from approximately 10^2^ to 0 viral RNA copies/µL (Table 3) were tested using twelve replicates each. Cq values ≥ 40 were considered negative. The lower limit of detection was defined as the concentration of viral copies achieving a 95% positivity hit rate (LOD95). The LOD95 was calculated with probit regression analysis using IBM SPSS software version 24.

**Table 2.** CHIKV and ONNV strains used in this study and viral strains of specificity panel.

Genus	Virus species and acronyms		Strain	Viral load (TCID_50_/mL)	Reference on EVAg or NCPV catalogues
*Alphavirus*	Chikungunya virus	CHIKV (ECSA 1)	UVE/CHIKV/UNK/XX/ROSS	10 ^6.49^	001v-EVA1455 (EVAg)
* *	Chikungunya virus	CHIKV (ECSA 2)	UVE/CHIKV/2011/CD/Brazza_MRS1	10 ^6.57^	001v-EVA960 (EVAg)
* *	Chikungunya virus	CHIKV (ECSA 3)	UVE/CHIKV/2006/RE/LR2006_OPY1	10 ^7.07^	001v-EVA83 (EVAg)
* *	Chikungunya virus	CHIKV (West African)	UVE/CHIK/1983/SN/WA 37997	10 ^6.22^	001V-02448 (EVAg)
* *	Chikungunya virus	CHIKV (Asian)	H20235/STMARTIN/2013	10 ^7.07^	001v-EVA1540 (EVAg)
* *	O’nyong-nyong virus	ONNV	UVE/ONNV/UNK/SN/Dakar 234	10 ^4.22^	001v-EVA1044 (EVAg)
* *	Venezuelan equine encephalitis virus	VEEV	UVE/VEEV/UNK/XX/TC83 vaccine	10 ^9.42^	001v-EVA1459 (EVAg)
* *	Western equine encephalitis virus	WEEV	UVE/WEEV/UNK/XX/47a	10 ^8.32^	001v-EVA1479 (EVAg)
* *	Eastern equine encephalitis virus	EEEV	UVE/EEEV/1999/XX/H178_99	10 ^7.82^	001v-EVA1480 (EVAg)
* *	Ross River virus	RRV	5281v	10 ^8.16^	0005281v (NCPV)
* *	Mayaro virus	MAYV	UVE/MAYV/1954/TT/TC625	10 ^8.82^	001v-EVA502 (EVAg)
* *	Semliki Forest virus	SFV	UVE/SFV/UNK/XX/1745	10 ^4.42^	001V-02468 (EVAg)
* *	Sindbis virus	SINV	UVE/SINV/UNK/EG/Egypt 339	10 ^4.32^	001V-02469 (EVAg)
*Flavivirus*	Dengue virus-1	DENV-1	1579/18	10 ^7.08^	007V-03123 (EVAg)
* *	Dengue virus-2	DENV-2	UVE/DENV-2/1998/MQ/H_ IMTSSA-MART_98-703	10 ^4.57^	001v-EVA1019 (EVAg)
* *	Dengue virus-3	DENV-3	UVE/DENV-3/UNK/PH/H87	10 ^6.42^	001v-EVA242 (EVAg)
* *	Dengue virus-4	DENV-4	UVE/DENV-4/2012/BR/4829	10 ^6.07^	001V-03361 (EVAg)
* *	Japanese encephalitis virus	JEV	UVE/JEV/2009/LA/CNS769	10 ^5.57^	001V-02217 (EVAg)
* *	Saint Louis encephalitis virus	SLEV	UVE/SLEV/UNK/US/MSI-7	10 ^4.82^	001v-EVA128 (EVAg)
* *	Tick-borne encephalitis virus	TBEV	UVE/TBEV/2013/FR/32.11 WT-PCR	10 ^8.82^	001V-02352 (EVAg)
* *	Zika virus	ZIKV (Asian lineage)	UVE/ZIKV/1947/UG/MR766	10 ^4.32^	001v-EVA143 (EVAg)
* *	Zika virus	ZIKV (African lineage)	H/PF/2013	10 ^6.57^	001v-EVA1545 (EVAg)
* *	Yellow Fever virus	YFV	UVE/YFV/UNK/XX/Vaccinal strain 17D	10 ^4.32^	001v-EVA67 (EVAg)
* *	West Nile virus	WNV	UVE/WNV/2008/US/R94224	10 ^7.32^	001V-02224 (EVAg)
* *	Usutu virus	USUV	UVE/USUV/1959/ZA/SAAR-1776	10 ^5.32^	001v-EVA138 (EVAg)
* *	Murray Valley encephalitis virus	MVEV	UVE/MVEV/UNK/AU/3329	10 ^4.32^	001v-EVA145 (EVAg)
*Phlebovirus*	Toscana virus	TOSV	UVE/TOSV/2014/FR/5904	10 ^7.42^	001V-02461 (EVAg)

**Table 3.** Limits of detection of the ONNV RT-qPCR monoplex assay and the ONNV-CHIKV duplex RT-qPCR assay.

		ONNV monoplex assay				ONNV-CHIKV duplex assay			
	Viral RNA copies/µL	Total samples tested, No.	Positive samples, No.	Cq, Mean [SD]	LOD95 [CI]	Total samples tested, No.	Positive samples, No.	Cq, Mean [SD]	LOD95 [CI]
ONNV strain	76	12	12/12	32.2 [0.1]	1.5 copies/µL [1.1-2.9]	12	12/12	32.2 [0.1]	4.3 copies/µL [3.4-6.3]
	15.2	12	12/12	34.7 [0.3]		12	12/12	34.7 [0.3]	
	7.6	12	12/12	35.7 [0.3]		12	12/12	35.7 [0.5]	
	3.8	12	12/12	37.5 [1.0]		12	10/12	36.7 [0.5]	
	1.9	12	12/12	37.8 [0.8]		12	6/12	38.3 [0.9]	
	1.0	12	9/12	38.8 [0.6]		12	3/12	39.0 [0.9]	
	0.5	12	7/12	39.2 [0.5]		12	0/12		
	0.2	12	1/12	39.3		12	0/12		
CHIKV – ECSA 1 strain	39	-				12	12/12	34.5 [0.3]	2.1 copies/µL [1.6-3.3]
	7.8					12	12/12	36.6 [0.4]	
	3.9					12	12/12	37.3 [0.5]	
	2.0					12	11/12	38.3 [0.7]	
	1.0					12	7/12	38.6 [0.8]	
	0.5					12	3/12	39.1 [0.1]	
	0.2					12	3/12	38.4 [1.0]	
	0.1					12	1/12	37.2	
CHIKV – ECSA 2 strain	45.2	-				12	12/12	33.4 [0.2]	1.1 copies/µL [0.9-1.8]
	9.0					12	12/12	35.4 [0.3]	
	4.5					12	12/12	36.3 [0.3]	
	2.3					12	12/12	37.5 [0.6]	
	1.1					12	11/12	37.8 [0.6]	
	0.6					12	11/12	38.4 [0.4]	
	0.3					12	2/12	37.7 [0.6]	
	0.1					12	1/12	39.0	
CHIKV – ECSA 3 strain	74.0	-				12	12/12	34.0 [0.2]	3.5 copies/µL [2.6-5.8]
	14.8					12	12/12	36.0 [0.3]	
	7.4					12	12/12	37.0 [0.6]	
	3.7					12	11/12	37.8 [0.5]	
	1.9					12	9/12	38.6 [0.5]	
	0.9					12	6/12	38.8 [0.3]	
	0.5					12	3/12	39.6 [0.1]	
	0.2					12	1/12	38.6 [1.9]	
CHIKV – West African strain	59.3	-				12	12/12	34.2 [0.1]	3.6 copies/µL [2.7-6.1]
	11.9					12	12/12	36.4 [0.3]	
	5.9					12	12/12	37.5 [0.8]	
	3.0					12	10/12	38.4 [0.7]	
	1.5					12	7/12	38.1 [1.7]	
	0.7					12	5/12	39.1 [1.1]	
	0.4					12	1/12	38.5	
	0.2					12	2/12	39.6 [0.1]	
CHIKV – Asian strain	66.7	–				12	12/12	33.9 [0.1]	2.8 copies/µL [1.9-7.0]
	13.3					12	12/12	36.1 [0.3]	
	6.7					12	12/12	37.0 [0.6]	
	3.3					12	12/12	38.0 [0.4]	
	1.7					12	10/12	38.6 [0.7]	
	0.8					12	7/12	39.5 [0.4]	
	0.4					12	7/12	38.9 [0.6]	
	0.2					12	5/12	39.4 [0.2]	

### Linearity

The linear range of ONNV and CHIKV monoplex assays and of the duplex ONNV-CHIKV assay was assessed using ten-fold serial dilutions with concentrations ranging approximately from 10^8^ to 10^2^ viral RNA copies/µL of ONNV and CHIKV strains. Two replicates of each dilution were tested in a single run. Linear correlation coefficient (R^2^) and qPCR efficiency were calculated.

### Specificity

The specificity of the ONNV monoplex assay and the ONNV-CHIKV duplex assay was evaluated against a panel of 21 related and unrelated viruses from *Alphavirus* (n = 7), *Flavivirus* (n = 13) and *Phlebovirus* (n = 1) genera. The ONNV monoplex assay was additionally tested against the five CHIKV strains representing CHIKV. All viral strains included in the specificity panel were provided by the European Virus Archive goes Global (EVAg, https://www.european-virus-archive.com/) except Ross River virus, which was provided by the National Collection of Pathogenic Viruses (NCPV, https://www.pheculturecollections.org.uk/collections/ncpv.aspx). The specificity panel of viral strains is presented in Table 2.

### Clinical validation

A total of 47 clinical samples tested positive for CHIKV RNA at the National Reference Center for Arboviruses (Marseille, France) were kindly provided for comparison using the ONNV-CHIKV duplex assay and the Panning CHIKV monoplex assay [12]. Because ONNV-positive clinical samples were not available for testing, qualified-negative plasma provided by the French Blood Bank was spiked with ONNV strain in ten-fold serial dilutions in order to simulate clinical samples with different viral loads. Four replicates were tested in parallel using the ONNV monoplex assay and the ONNV-CHIKV duplex assay, using the same RT-qPCR conditions.

Additionally, to ensure that the ONNV-CHIKV duplex does not react with other pathogens we tested 36 sera from febrile patients of the Assistance Publique Hôpitaux de Marseille with an initial suspicion of CHIKV that then tested PCR negative for CHIKV [13]. Twenty-three samples were found positive for other pathogens (*plasmodium falciparum* (n = 10), cytomegalovirus (n = 4), Epstein Barr virus (n = 7), hepatitis A virus (n = 2)), while for 13 no etiological agent was identified. These samples were stored at −20°C or −40°C after collection. The ethics committee of Assistance Publique Hopitaux de Marseille approved this study under registration PADS23-93.

## Results

### Design of ONNV monoplex RT-qPCR assay and development of ONNV-CHIKV duplex RT-qPCR assay

**Figure 1.**
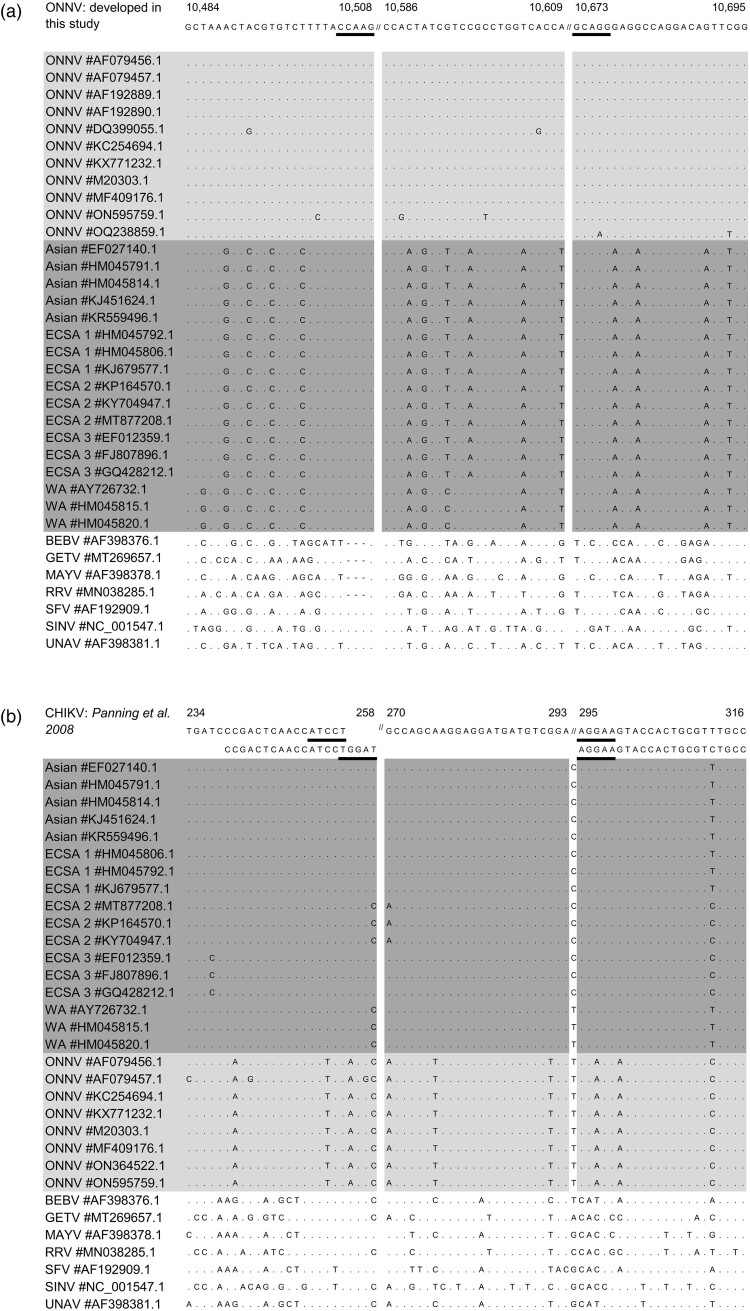
*In silico* analysis of the ONNV monoplex assay (A) and CHIKV monoplex assay included in the ONNV-CHIKV duplex assay (B). In dark grey, selected CHIKV strains; in light grey, relevant available ONNV sequences; in white, other closely related *Alphavirus*. GenBank Accession number of reference sequences: M20303 (A) and MG280943 (B). The five 3’ nucleotides of primers are underlined because mismatches in these positions are of particular relevance for assay sensitivity.

Genome sequences of ONNV, CHIKV and other phylogenetically-close alphaviruses were aligned to perform an *in silico* analysis of the ONNV monoplex assay, as well as to evaluate the Panning CHIKV monoplex assay selected to be included in the ONNV-CHIKV duplex assay [12]. Mismatches between this panel of sequences and primers and probe sets were checked, giving specific attention to the five 3’ terminal nucleotides of primers, since mismatches in these positions are of particular relevance for assay sensitivity [10]. A conserved region of the envelope gene was selected as target for the ONNV monoplex assay. The *in silico* analysis highlighted several mismatches between ONNV primers and probe and strains of alphaviruses other than ONNV. Results of this *in silico* analysis are presented in Figure 1.

### Limit of detection

**Figure 2.**
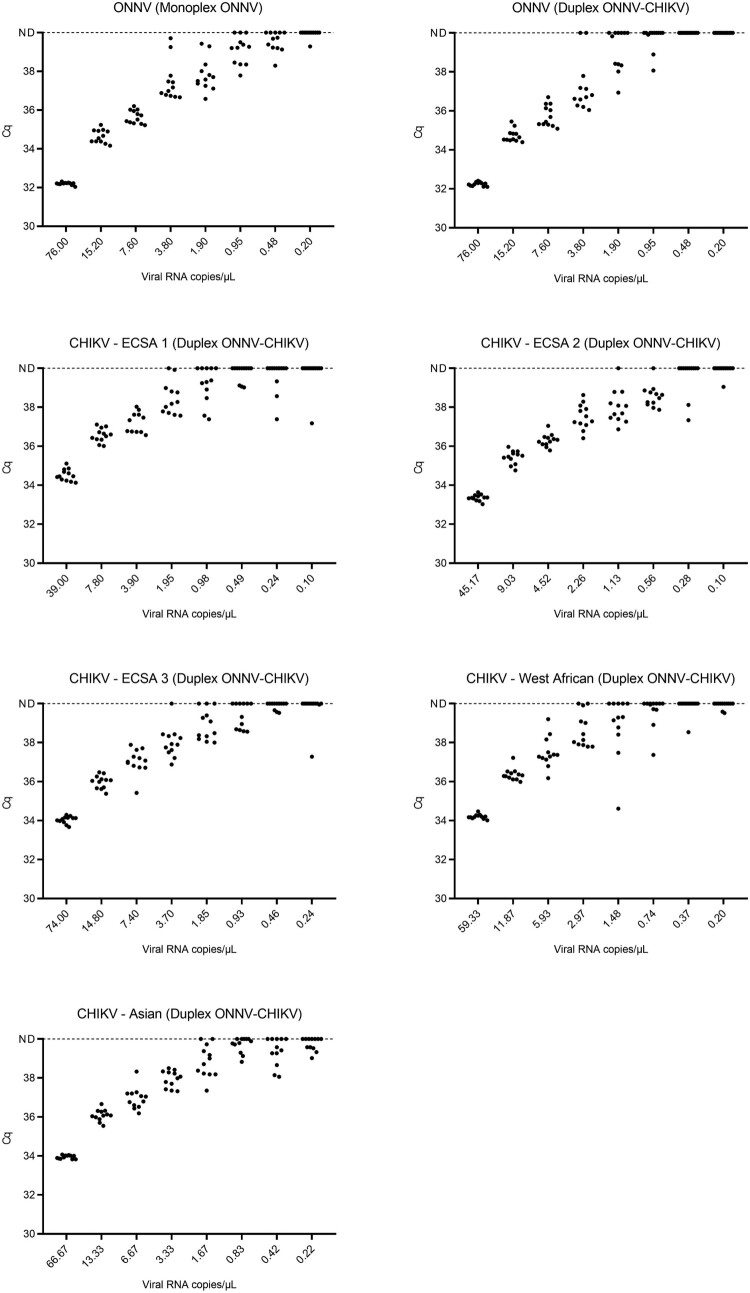
Detection of dilutions of one ONNV strain and five CHIKV strains with the ONNV RT-qPCR monoplex assay and the ONNV-CHIKV duplex RT-qPCR assay. ND: not detected.

The LOD95 of the ONNV monoplex assay was 1.5 RNA copies/µL [95% CI, 1.0-2.5]. LOD of the ONNV-CHIKV duplex assay was 4.3 RNA copies/µL [95% CI, 3.4-6.3] for ONNV, 2.1 RNA copies/µL [95% CI, 2.6-3.4] for CHIKV ECSA 1 strain, 1.1 RNA copies/µL [95% CI, 0.8-1.7] for CHIKV ECSA 2 strain, 3.5 RNA copies/µL [95% CI, 2.6-5.8] for CHIKV ECSA 3 strain, 3.6 RNA copies/µL [95% CI, 2.7-6.2] for CHIKV West African strain and 2.8 RNA copies/µL [95% CI, 1.9-7.0] for Asian strain (Table 3 and Figure 2).

### Linearity

**Figure 3.**
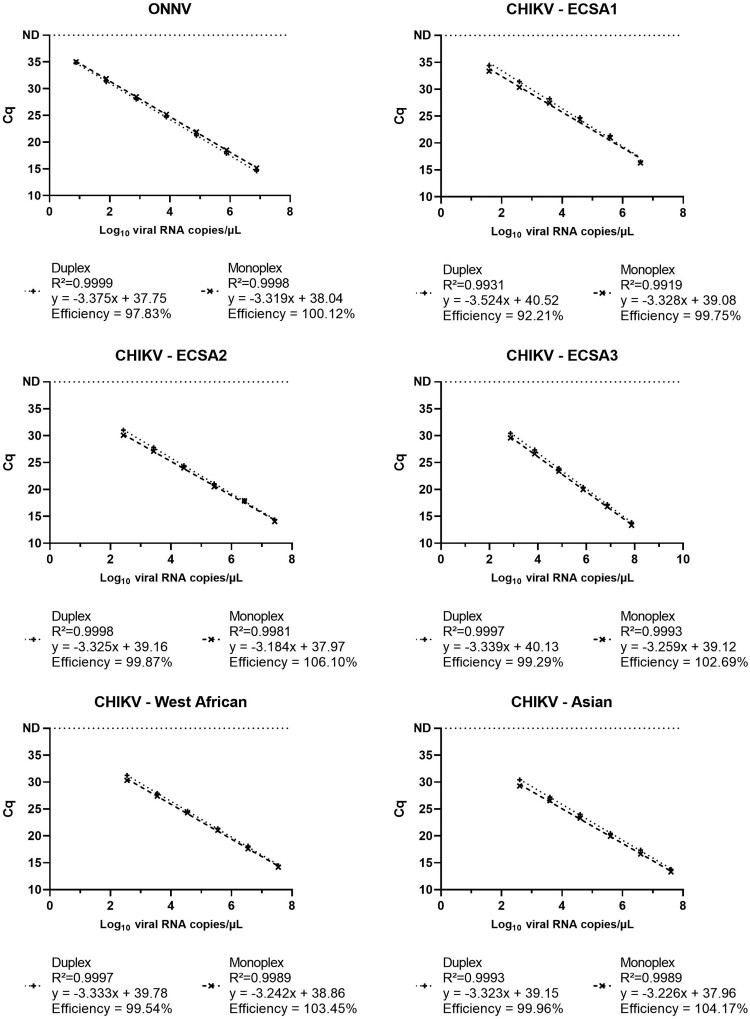
Linear range of the duplex ONNV-CHIKV assay and the monoplex assays included in the duplex format. Each dilution was tested in duplicate in a single run and mean Cq is represented in the figure.

The duplex assay and both monoplex assays included in the duplex format were found to be linear within the tested concentration range from approximately from 10^8^ to 10^2^ viral RNA copies/µL of ONNV and CHIKV strains with *R*^2^ > 0.99 and qPCR efficiencies ranging from 92.21% to106.10%. Results of the linearity test are presented in Figure 3. The assays are thus proven to have consistent efficiency of amplification within the desired range of 90-100% [14]. While it is not relevant for arboviruses like CHIKV and ONNV to quantify viral loads via PCR, the results of this test indicate this could be achieved using this test. However, this would require further evaluation of the assay.

### Specificity

Both the ONNV monoplex and the ONNV-CHIKV duplex assay only detected the homologous positive controls and did not react with other tested viruses, namely Venezuelan equine encephalitis virus, Western equine encephalitis virus, Eastern equine encephalitis virus, Ross River virus, Mayaro virus, Semliki Forest virus, Sindbis virus, Dengue virus 1-4, Japanese encephalitis virus, Saint Louis encephalitis virus, Tick-borne encephalitis virus, Zika virus (Asian lineage), Zika virus (African lineage), Yellow Fever virus (vaccinal strain 17D), West Nile virus, Usutu virus, Murray Valley encephalitis virus, Toscana virus (for details see Table 2). Thus, the ONNV monoplex assay proved to be 100% specific for ONNV and the ONNV-CHIKV duplex assay proved to be 100% specific for CHIKV (FAM channel) and ONNV (VIC channel). Moreover, no amplification was observed in potentially cross-reactive samples when tested using the RT-qPCR ONNV monoplex and ONNV-CHIKV multiplex assay.

### Clinical validation

Among the 47 clinical CHIKV-positive samples tested, 46 were positive when tested with Panning CHIKV monoplex assay. The duplex ONNV-CHIKV assay could detect the same 46 samples and one additional, weakly positive, sample (Cq value 37.1). Twenty-one out of forty-six samples provided a lower Cq value with the ONNV-CHIKV duplex compared to CHIKV monoplex assay. The ΔCq values (Cq value of Panning CHIKV monoplex assay – Cq value of ONNV-CHIKV duplex assay) was generally low, with ΔCq > 1 only observed at Cq > 30. The results of molecular testing of clinical CHIKV-positive samples are presented in Table S3. No clinical ONNV-positive samples were available since acute cases are very rarely reported, we thus used ONNV-spiked plasma. Both the monoplex ONNV assay and the duplex ONNV-CHIKV assay detected 100% of samples, with small differences between Cq values (highest ΔCq value between ONNV-CHIKV duplex and ONNV monoplex assay was 0.3). Results are presented in Table S4.

ONNV-CHIKV duplex assay was also negative in all 36 sera from patients suspected of CHIKV but infected by other pathogens or in whom no etiologic agent was identified.

## Discussion

O’nyong-nyong virus and, to a lesser degree, chikungunya virus, are neglected pathogens in Africa. A study has recently shown that in Mali, a country where ONNV acute cases have never been officially reported and CHIKV has been sporadically detected, ONNV estimated prevalence was even higher than CHIKV (30% and 13%, respectively) [11], suggesting an underreported circulation that probably occurs in several other countries.

While for CHIKV the recent occurrence of extensive epidemics has encouraged investigations of viral circulation in Africa and has impelled laboratories to develop specific detection assays, the situation is different for ONNV. Its contribution to the burden of febrile illnesses in Africa is actually unknown due to lack of surveillance and sub-optimal diagnostic tools. Few in-house PCR assays for the detection of ONNV have been described so far [15–18]. The EQA conducted by EVD-LabNet represented a useful occasion for participating laboratories to assess their ability to correctly detect, identify, characterize and diagnose ONNV and highlighted the need for better molecular detection assays.

Little data is available to describe the viremic window, the viremia levels, or the best biological sample to use for ONNV diagnosis; therefore, ONNV molecular tests may prove extremely useful not only for viral detection at acute phase but also to better understand the natural course of infection. Nonetheless, since the viremic window is yet to be fully described, molecular assays should be used in complement to serological assays. In this study, we described the design and evaluation of a new RT-qPCR assay for ONNV that demonstrates good analytical performances when used in monoplex format or as a component of a ONNV–CHIKV duplex assay. To avoid amplification of other pathogens we designed the assay to present several important mismatches to other close relatives of ONNV. Additionally, specificity was tested against a wide panel of other arboviruses and other pathogens. In both forms, the assay proved 100% specific with no unspecific amplification. This multiplex format is particularly useful in vast areas of Africa where ONNV and CHIKV co-circulate. First, because the two viruses cause an overlapping clinical syndrome, the etiological agent is thus impossible to determine without laboratory tests. Second, multiplex format allows screening of two suspected pathogens in a single PCR run, presenting important advantages in regions with limited diagnostic capacity, with a significant decrease of costs, time and efforts.

This study has some limitations. Due to the scarce availability of clinical ONNV samples we used spiked plasma samples for the clinical evaluation, and the ONNV assay showed excellent analytical performance in both monoplex and duplex form. It is common practice to evaluate and validate molecular assays using spiked samples and, in our experience, spiked samples are good surrogates for clinical samples during the validation of molecular assays [19]. Still, the performance of the ONNV assay in monoplex and duplex format will have to be confirmed in the field.

The design of the PCR assay (both monoplex and duplex assay) for detection of ONNV was limited by the small number of sequences available on GenBank, which also limits molecular epidemiology studies. For the analytical evaluation we used strains representing the circulating genetic diversity of both CHIKV (n = 5) and ONNV (n = 1). Molecular pathogen detection assays need to be updated regularly to ensure effective detection of currently circulating strains. In case of emergence of a new strain, a new primer or probe can be added to the existing assay.

It is plausible that this assay will contribute to the identification of new ONNV cases and to the extension of genomic data collection. Identification of eventual new ONNV strains may require the design of improved molecular assays in the future. Actually, this assay proves to be a useful diagnostic tool for the accurate detection of ONNV (and CHIKV when used in multiplex format), thus helping to investigate etiology of febrile illnesses in Africa.

## Supplementary Material

ONNV CHIKV Supplementary data.docx
