# Supplementary material for: Analytical and clinical evaluation of a duplex RT-qPCR assay for the detection and identification of o’nyong-nyong and chikungunya virus
Source: Emerg Microbes Infect. 2024 Nov 12;13(1):2429650. doi: 10.1080/22221751.2024.2429650 (PMC11587735; doi:10.1080/22221751.2024.2429650)
Supplement: ONNV CHIKV Supplementary data.docx [file TEMI_A_2429650_SM7726.docx]

Supplementary Materials

**Analytical and clinical evaluation of a duplex RT-qPCR assay for the detection and identification of o’nyong-nyong and chikungunya virus**

Konrad M. Wesselmann^a*^, Lea Luciani^a,b^, Laurence Thirion^a^, Xavier de Lamballerie^a,b,c^, Remi Charrel^a,b¶^, Laura Pezzi^a,b¶^

^a^Unité des Virus Émergents (UVE: Aix-Marseille Univ, Università di Corsica, IRD 190, Inserm 1207, IRBA), France

^b^Assistance publique-hôpitaux de Marseille (AP-HM), Service de virologie aiguë et tropicale, Marseille, France

^c^Centre National de Référence des Arbovirus, Inserm-IRBA, Marseille, France

^¶^ Equal contribution

*Address correspondence to Konrad M Wesselmann: [konrad.wesselmann@univ-amu.fr](mailto:konrad.wesselmann@univ-amu.fr)

Table S 1: Accession numbers of excluded sequences and reason.

| LY683326.1 | Not referring to genomic RNA |
| --- | --- |
| NC_001512.1 | Identical to M20303 |
| NC_075006.1 | Identical to AF079456.1 |
| OF092881.1 | Not referring to genomic RNA |
| GN354779.1 | Not referring to genomic RNA |
| GN354780.1 | Not referring to genomic RNA |
| GN354781.1 | Not referring to genomic RNA |
| GN354782.1 | Not referring to genomic RNA |
| GN354783.1 | Not referring to genomic RNA |
| GN354784.1 | Not referring to genomic RNA |
| GN354785.1 | Not referring to genomic RNA |
| GN354786.1 | Not referring to genomic RNA |
| GN354787.1 | Not referring to genomic RNA |
| GN354788.1 | Not referring to genomic RNA |

Table S 2: Accession numbers of sequences used for the design of ONNV RT-qPCR assay and mismatch analysis (Figure 1).

| **CHIKV** | **ONNV** | **Other alphaviruses** |
| --- | --- | --- |
| Asian #EF027140.1 | ONNV #AF192890 | BEBV #AF398376.1 |
| Asian #HM045791.1 | ONNV #AF192889 | GETV #MT269657.1 |
| Asian #HM045814.1 | ONNV #AF079457 | MAYV #AF398378.1 |
| Asian #KJ451624.1 | ONNV #MF409176 | RRV #MN038285.1 |
| Asian #KR559496.1 | ONNV #AF079456 | SFV #AF192909.1 |
| ECSA 1 #HM045806.1 | ONNV #KX771232 | SINV #NC_001547.1 |
| ECSA 1 #HM045792.1 | ONNV #DQ399055 | UNAV #AF398381.1 |
| ECSA 1 #KJ679577.1 | ONNV #DQ383273 |  |
| ECSA 2 #MT877208.1 | ONNV #DQ383272 |  |
| ECSA 2 #KP164570.1 | ONNV #DQ381540 |  |
| ECSA 2 #KY704947.1 | ONNV #M20303 |  |
| ECSA 3 #EF012359.1 | ONNV #OQ238859 |  |
| ECSA 3 #FJ807896.1 | ONNV #ON364522 |  |
| ECSA 3 #GQ428212.1 | ONNV #KC254694 |  |
| WA #AY726732.1 | ONNV #ON595759 |  |
| WA #HM045815.1 |  |  |
| WA #HM045820.1 |  |  |

Table S 3 : CHIKV positive clinical samples, tested with Panning CHIKV monoplex assay and ONNV-CHIKV duplex assay. Samples #1 to #7 have been diluted to simulate weakly positive samples.

| **Sample ID** | **Cq value (ONNV-CHIKV duplex)** | **Cq value (Panning CHIKV monoplex assay)** | **ΔCq value (Cq value ONNV-CHIKV duplex – Cq value Panning CHIKV monoplex assay)** |
| --- | --- | --- | --- |
| #1 | 35.5 | 35.8 | -0.3 |
| #2 | 36.9 | 38.6 | -1.7 |
| #3 | 37.0 | 39.5 | -2.5 |
| #4 | 34.3 | 34.3 | 0.0 |
| #5 | 37.7 | 37.8 | 0.1 |
| #6 | 37.1 | ND | - |
| #7 | 36.4 | 35.4 | 1.0 |
| #8 | 22.2 | 22.2 | 0.0 |
| #9 | 24.1 | 24.2 | -0.1 |
| #10 | 24.1 | 24.2 | -0.1 |
| #11 | 22.8 | 22.9 | 0.1 |
| #12 | 24.0 | 24.0 | 0.0 |
| #13 | 22.4 | 22.5 | -0.1 |
| #14 | 24.3 | 24.2 | 0.1 |
| #15 | 24.1 | 23.9 | 0.2 |
| #16 | 24.3 | 24.3 | 0.0 |
| #17 | 21.3 | 21.1 | 0.2 |
| #18 | 24.0 | 23.8 | 0.2 |
| #19 | 21.8 | 21.9 | -0.1 |
| #20 | 24.0 | 23.7 | 0.3 |
| #21 | 20.5 | 20.8 | -0.3 |
| #22 | 25.1 | 25.6 | -0.5 |
| #23 | 22.8 | 22.2 | 0.6 |
| #24 | 22.0 | 22.0 | 0.0 |
| #25 | 24.0 | 23.7 | 0.3 |
| #26 | 25.1 | 25.2 | -0.1 |
| #27 | 23.3 | 23.3 | 0.0 |
| #28 | 22.0 | 22.0 | 0.0 |
| #29 | 18.3 | 18.2 | 0.1 |
| #30 | 21.0 | 21.1 | -0.1 |
| #31 | 21.1 | 21.1 | 0.0 |
| #32 | 18.7 | 18.7 | 0.0 |
| #33 | 18.0 | 17.5 | 0.5 |
| #34 | 20.8 | 20.5 | 0.3 |
| #35 | 19.5 | 19.4 | 0.1 |
| #36 | 17.2 | 17.4 | -0.2 |
| #37 | 18.3 | 18.4 | 0.1 |
| #38 | 19.9 | 19.9 | 0.0 |
| #39 | 20.1 | 20.2 | 0.1 |
| #40 | 20.2 | 20.2 | 0.0 |
| #41 | 19.2 | 19.2 | 0.0 |
| #42 | 18.2 | 17.5 | 0.7 |
| #43 | 19.2 | 19.2 | 0.0 |
| #44 | 17.3 | 17.4 | -0.1 |
| #45 | 19.6 | 19.6 | 0.0 |
| #46 | 19.1 | 19.0 | 0.1 |
| #47 | 20.1 | 20.1 | 0.0 |

Table S 4: ONNV-spiked plasma samples tested with ONNV monoplex and ONNV-CHIKV duplex assay.

| **Dilution** | **Average Cq value ONNV-CHIKV duplex** | **Average Cq value ONNV monoplex** | **ΔCq value (Cq value** **ONNV-CHIKV duplex – Cq value ONNV monoplex assay)** |
| --- | --- | --- | --- |
| E-3 | 22.2 | 22.1 | 0.1 |
| E-4 | 25.8 | 25.5 | 0.3 |
| E-5 | 29.2 | 29.0 | 0.2 |
| E-6 | 32.6 | 32.4 | 0.2 |
| E-7 | 35.4 | 35.5 | -0.1 |
